# Disaster exposure as a risk factor for mental health problems, eighteen months, four and ten years post-disaster – a longitudinal study

**DOI:** 10.1186/1471-244X-12-147

**Published:** 2012-09-18

**Authors:** Bellis van den Berg, Albert Wong, Peter G van der Velden, Hendriek C Boshuizen, Linda Grievink

**Affiliations:** 1 Regioplan Policy Research, Nieuwezijds Voorburgwal 35, Amsterdam, RD, 1012, the Netherlands; 2 National Institute for Public Health and the Environment (RIVM), A. van Leeuwenhoeklaan 9, Bilthoven, BA, 3720, The Netherlands; 3 Institute for Psychotrauma, Nienoord 5, Diemen, XE, 1112, the Netherlands; 4 INTERVICT, Tilburg University, Tilburg, the Netherlands; 5 National Institute for Public Health and the Environment (RIVM), A. van Leeuwenhoeklaan 9, Bilthoven, BA, 3720, The Netherlands; 6 National Institute for Public Health and the Environment (RIVM), A. van Leeuwenhoeklaan 9, Bilthoven, BA, 3720, The Netherlands

**Keywords:** Disasters, Longitudinal studies, Risk Factors, Stress, Psychological, Stress Disorders, Post-Traumatic

## Abstract

**Background:**

Disaster experiences have been associated with higher prevalence rates of (mental) health problems. The objective of this study was to examine the independent relation between a series of single disaster experiences versus the independent predictive value of a accumulation of disaster experiences, i.e. a sum score of experiences and symptoms of distress and post-traumatic stress disorder (PTSD).

**Methods:**

Survivors of a fireworks disaster participated in a longitudinal study and completed a questionnaire three weeks (wave 1), eighteen months (wave 2) and four years post-disaster (wave 3). Ten years post-disaster (wave 4) the respondents consisted of native Dutch survivors only. Main outcome measures were general distress and symptoms of PTSD.

**Results:**

Degree of disaster exposure (sum score) and some disaster-related experiences (such as house destroyed, injured, confusion) were related to distress at waves 2 and 3. This relation was mediated by distress at an earlier point in time. None of the individual disaster-related experiences was independently related to symptoms of distress. The association between the degree of disaster exposure and symptoms of PTSD at waves 2 and 3 was still statistically significant after controlling for symptoms of distress and PTSD at earlier point in time. The variable ‘house destroyed’ was the only factor that was independently related to symptoms of PTSD at wave 2. Ten years after the disaster, disaster exposure was mediated by symptoms of PTSD at waves 2 and 3. Disaster exposure was not independently related to symptoms of PTSD ten years post-disaster.

**Conclusions:**

Until 4 years after the disaster, degree of exposure (a sum score) was a risk factor for PTSD symptoms while none of the individual disaster experiences could be identified as an independent risk factor. Ten years post-disaster, disaster exposure was no longer an independent risk factor for symptoms of PTSD. Since symptoms of PTSD and distress at earlier waves perpetuate the symptoms at later waves, health care workers should aim their resources at those who still have symptoms after one and a half year post-disaster, to prevent health problems at medium and long-term.

## Background

Each year disasters affect large numbers of people throughout the world. These traumatic experiences may result in a wide range of mental and physical health consequences [1,2]. Insight into factors that predict mental health problems among survivors enables health care workers and policy makers to estimate the number of people that develop psychopathology and to guide (early) interventions [3,4]. Screening survivors for disaster exposure immediately after the disaster is important for two reasons. Firstly, disaster-related emotions and experiences are subject to recall bias and collecting information about disaster-related experiences shortly after the disaster may prevent recall bias as much as possible. Secondly, disaster-related experiences such as property damage and physical injury as well as initial post-traumatic stress reactions predict health problems in the longer term [5,6]. Knowledge of factors that predict psychological problems can be used for (mental) health triage.

The association between disaster exposure and psychological problems, such as anxiety, depression and PTSD, has frequently been studied, showing a positive relation [1,7-9]. Since most of these studies were performed in the months or years post-disaster, recall bias could, however, not be excluded. Also, in most previous studies the independent predictive value of disaster exposure was not examined by using multivariate models. Moreover, many studies examined the degree of disaster exposure by adding different disaster experiences into one single factor [1,7,8,10-12]. For example, in a national U.S. study on the WTC disaster, the factor September 11-related loss was composed of different types of losses, such as loss of property, injury and death of someone close [12]. Although these studies show that the degree of disaster exposure is related to higher prevalence rates of psychological problems [1,10,11], it is not clear from these previous studies whether specific experiences are responsible for a higher number of symptoms.

In the current study we extend previous research by examining the independent predictive value of disaster exposure in two ways. We compare the independent predictive value of twelve different disaster experiences versus the independent predictive value of a accumulation of disaster experiences, i.e. a sum score of experiences. Since an accumulation of disaster experiences may reflected an (actual) accumulation of sources of stress or resources lost during the disaster, we expected that a sum score would better independently predict post-event mental health problems (symptoms of PTSD and distress) than a series of single experiences of factors [13]. In this study we use a longitudinal dataset collected after the Enschede fireworks disaster in the Netherlands. To our knowledge, this is the first longitudinal study that was performed a few weeks until ten years post-disaster.

## Methods

### Participants and procedure

On 13 May 2000 a fireworks depot exploded in a residential area in the city of Enschede, the Netherlands. As a result of the explosion, 23 people were killed, more than 900 people were injured and 500 houses were severely damaged or destroyed. After the disaster, the Dutch Ministry of Health, Welfare and Sports initiated a longitudinal study into the health consequences of the disaster. An initial survey was commenced within three weeks after the disaster (wave 1). All adult residents were invited to complete the questionnaire by means of letters and announcements in the local media. In total, 1567 survivors completed the questionnaire at wave 1 (estimated response rate ≈ 30%) [14].

Approximately eighteen months after the disaster, from November 2001 to January 2002, a second wave was performed. All participants at wave 1 who had given written informed consent for future contact received an announcement letter. In total, 1116 survivors (response 72%) completed a questionnaire at wave 2 [15].

Nearly four years post-disaster (January – March, 2004) a third wave was performed. Except for participants who were lost to follow-up, all survivors of wave 1 were invited to complete a questionnaire. In total, 995 survivors (response 66%) completed a questionnaire at wave 3. Details of the study population and procedures have been described elsewhere [15-17].

Recently, a fourth wave was performed among native Dutch survivors, ten years post-disaster. At previous waves we put much effort in including immigrant survivors into the study by means of telephone calls and house visits. Because of limited budget we only focused on the native Dutch survivors at the fourth wave. In total, 826 native Dutch survivors could be invited to complete a questionnaire. Eventually, 594 native Dutch survivors completed a questionnaire at wave 4 (response rate 72%).

### Measures

The questionnaires primarily included scales that had been previously validated in the Dutch population, as described below.

#### Symptoms of distress and PTSD

Symptoms of distress were measured at waves 1, 2 and 3 using the Dutch version of the Symptom Checklist-90-R (SCL-90-R) [18]. The SCL-90-R asks for a broad range of psychological problems and symptoms of psychopathology during the past week. The SCL-90-R consist of nine sub-scales measuring symptoms of somatisation, obsessive-compulsive symptoms, symptoms of interpersonal sensitivity, depression, anxiety, hostility, phobic anxiety, paranoid ideation and psychoticism. In the present study, results are presented for the total score on the SCL-90-R ranging from 90 to 450. The validity and reliability of the Dutch SCL-90-R has been shown to be satisfactory. The internal consistency of the SCL-90-R was very good at the three waves (Cronbach’s α ≥ .89).

Disaster-related PTSD symptoms was assessed at all waves by using the Impact of Event Scale (IES) [19,20]. The IES measures symptoms of intrusive and avoidance in the past seven days , such as how often the respondent thought of the event, how often the respondent had dreams about the event, and how often pictures of the event popped into the respondents’ mind. The IES does not measure the PTSD symptoms of arousal or numbing. Scores on the fifteen items were rated on a 4-point Likert scale (0 = not at all, 1 = rarely, 3 = sometimes, 5 = often) in order to assess the degree of disaster-related intrusions and avoidance reactions, with total scores ranging from 0 to 75. The reliability and structure of the Dutch IES has proven to be adequate across various traumatic stressors. At all measurement points, the internal consistency was excellent (Cronbach’s α ≥ .94). The prevalence rates of distress and symptoms of PTSD at the first three waves have been described elsewhere [14-17,21].

#### Disaster-related factors

Several experiences during or soon after the disaster were recorded at wave 1: a relocation due to severely damaged or destroyed house; a loss of loved ones (family, colleagues, friends); injuries requiring medical treatment. In addition, several questions were asked about what survivors had seen, heard and felt during the disaster: ‘witnessed injured victims’, ‘witnessed deceased victims’, ‘heard yelling individuals’, ‘felt heart palpitations’, ‘felt intense anxiety’, ‘fled from explosions and/or fire’, ‘helped affected victims’, ‘felt feelings of guilt and/or shame’, and ‘a state of confusion’. To assess the degree of disaster exposure a scale was made of the twelve items (sum-score).

#### Background characteristics

The following demographic and lifestyle characteristics were measured: sex, age, educational level, occupational status (having a paid job), cigarette smoking, immigrant status (first and second generation, mainly of Turkish origin) and the number of chronic diseases, such as diabetes, cancer, asthma etc.

### Statistical analysis

To assess the relation between disaster exposure and psychological problems in the medium term, symptoms distress and symptoms of PTSD at waves 2 and 3 were considered as outcomes. To assess the impact of disaster exposure in the long term, symptoms of PTSD at wave 4 was used as an outcome.

Let *y*_*t*_ and *z*_*t*_ be the vectors containing scores for symptoms distress and symptoms of PTSD at wave *t* respectively. *y*_*t*_ and *z*_*t*_ with $t\in \left\{2,3\right\}$ were considered as outcome variables that reflect the degree of psychological problems in the medium term, while *z*_*4*_ was used as an outcome for psychological problems in the long run (*y*_*4*_ was not collected in our data). Furthermore, let *X* and *W* be design matrices containing confounders (including intercept) and disaster-related factors at *t*=1 respectively. Finally, let *d* be the degree of disaster exposure at *t*=1. A series of linear regression models were fitted to assess the relation between disaster exposure and symptoms of distress and PTSD terms at various waves *t* with$t\in \left\{2,3,4\right\}$. Given *t* the following models were estimated:

Model type 1 (degree of disaster exposure):

a. $y_{t}=X\gamma +d\kappa +\varepsilon$; $z_{t}=X\gamma +d\kappa +\varepsilon$

b. $y_{t}=X\gamma +d\kappa +y_{1}\alpha_{y1}+z_{1}\alpha_{z1}+\varepsilon$;

$\;z_{t}=X\gamma +d\kappa +y_{1}\alpha_{y1}+z_{1}\alpha_{z1}+\varepsilon$

c. $y_{t}=X\gamma +d\kappa +\sum_{i=1}^{2}y_{i}\alpha_{\mathrm{yi}}+\sum_{i=1}^{2}z_{i}\alpha_{\mathrm{zi}}+\varepsilon$;

$\;z_{t}=X\gamma +d\kappa +\sum_{i=1}^{2}y_{i}\alpha_{\mathrm{yi}}+\sum_{i=1}^{2}z_{i}\alpha_{\mathrm{zi}}+\varepsilon$

d. $z_{t}=X\gamma +d\kappa +\sum_{i=1}^{3}y_{i}\alpha_{\mathrm{yi}}+\sum_{i=1}^{3}z_{i}\alpha_{\mathrm{zi}}+\varepsilon$

Model type 2 (disaster related factors): These include the same model variants as in model type 1, except for model variant (d) ^a^ , with the difference that the disaster exposure term *dK* in all equations is replaced by the term *Wβ* describing the disaster-related factors.

α_·_ ,β, *y* and *K* are vectors of regression parameters within each respective model, and similarly, ∈ is the vector of residual errors within each respective model (and thus, each of these assume a different set of values within each model). Model variants (1d) were only estimated for *t*=4, while (1c) and (2c) were only estimated for $t\in \left\{3,4\right\}$.

In these models, the (demographic) variables sex, age, immigrant status, employed/unemployed, high/low educational level, smoking/non-smoking and number of chronic diseases measured at wave 1 were considered as potential confounders. We included smoking as a confounder because in an earlier study among survivors of the fireworks disaster smoking was a significant independent risk factor for psychological problems among survivors [22]. The number of chronic diseases was included as a confounder in all models because studies in the general population have shown that the chronically ill are relatively more at risk of health problems [23,24]. P-values are adjusted for multiple testing using the Benjamini-Hochberg method [25].

The confounders and disaster-related factors had missing values (up to about 20% for number of chronic diseases) (Table 1). To gauge the variability caused by this missingness, and also to minimise any potential bias that may arise due to missingness, we used Multiple Imputations by Chained Equations (MICE) [26]. All variables within the regression were included in the prediction matrix of the MICE procedure, as advocated by Van Buuren. Rubin (1987) suggests no more imputations are needed than five to ten. We generated ten imputations [27].

**Table 1:** Demographic characteristics and disaster-related factors measured at wave 1 (N = 1567)

	**%**	**% missing**
Male gender	47.1	0.06
Mean age	Mean = 42.1	0.2
Native Dutch	69.1	3.0
Paid job	55.5	3.2
High educational level	45.8	4.3
Smoking	38.3	2.9
≥ 1 Chronic diseases	29.9	19.7
House destroyed due to disaster	21.8	3.3
Injury self due to disaster	7.2	1.5
Lost a loved one due to disaster^a^	6.0	1.5
Witnessed injured victims^b^	67.3	0
Witnessed deceased victims	11.1	0
Heard yelling individuals^b^	59.9	0
Felt heart palpitations^b^	47.2	0
Felt intense anxiety^b^	62.2	0
Fled from explosions and/or fire^b^	63.2	0
Helped affected victims^b^	48.6	0
Feelings of guilt and/or shame	8.3	22.0
Confusion	39.6	15.8

^a^ Lost a family member, colleague or a friend. One percent of the survivors lost a family member (n = 19).

^b^ The item was coded as ‘no’ when it was not ticked.

To test the stability of the linear regression models, we ran several models in different steps (see models 1 to 4). Each regression model was estimated for each of the ten imputations, and the results were subsequently pooled. As such, the final results take into account both between-imputation and within-imputation variance.

We tested the risk of multicollinearity by studying the variance inflation factor [28]. Marquardt (1970) argued that this factor should not exceed 10. We found that the variance inflation factor was between 1 and 3 for each independent variable, suggesting that multicollinearity was not a severe issue [28].

## Results

### Disaster-related experiences of respondents at wave 1

The demographic characteristics and disaster exposure reported by the survivors are presented in Table 1. The majority of respondents reported disaster-related experiences such as ‘witnessed injured victims’ (67.3%), ‘heard yelling individuals’ (59.9%), and ‘fled from the explosion and/or fire’ (63.2%). A smaller number of respondents indicated that their houses had been destroyed (21.8%), that they were injured (7.2%) or that they had lost a loved one (6.0%).

### Association between disaster exposure and symptoms of distress among survivors at waves 2 and 3

Table 2 shows the results of the multiple linear regression analysis in which the relation between degree of disaster exposure (sum-score) and symptoms of distress at waves 2 and 3 was analyzed. The degree of disaster exposure (sum-score) was significantly associated with symptoms of distress at wave 2 (B = 5.43, SE = 0.65) and wave 3 (B = 5.16, SE = 0.68) (model 1). However, this association was no longer significant when symptoms of distress and PTSD at wave 1 and wave 2 were entered into the model (models 2 and 3). Symptoms of distress at waves 2 and 3 were positively associated with a higher number of symptoms of distress at an earlier point in time, but not to symptoms of PTSD at waves 1 and 2.

**Table 2:** **Associations between degree of disaster exposure and symptoms of distress at waves 2 and 3**^**a**^

	**Symptoms of distress at wave 2**						**Symptoms of distress at wave 3**								
	Model 1:			Model 2:			Model 1:			Model 2:			Model 3:		
	Disaster-related factors			Disaster-related factors and symptoms distress and PTSD at wave 1			Disaster-related factors			Disaster-related factors and symptoms distress and PTSD at wave 1			Disaster-related factors and symptoms of distress and PTSD at waves 1 and 2		
	B	SE	P	B	SE	P	B	SE	P	B	SE	P	B	SE	P
Intercept	46.03	12.85	< .01	47.37	11.70	< .01	41.22	13.06	< .01	43.35	12.18	< .01	12.37	9.92	n.s.
Degree of disaster exposure	5.43	0.65	< .001	0.39	0.71	n.s.	5.16	0.68	< .01	0.81	0.75	n.s.	0.46	0.60	n.s.
Symptoms of distress at wave 1				0.58	0.44	< .01				0.49	0.04	< .01	0.07	0.05	n.s.
Symptoms of PTSD at wave 1				−0.14	0.12	n.s.				−0.07	0.13	n.s.	0.0001	0.09	n.s.
Symptoms of distress at wave 2													0.70	0.05	< .01
Symptoms of PTSD at wave 2													0.12	0.13	n.s.

^a^ Multiple regression analysis, adjusted for sex, age, ethnicity, employment status, educational level, smoking/non-smoking and number of chronic diseases. N.s. = not significant.

Model 1, wave 2: Pooled R-Squared = 0.289, Pooled R-Squared C.I. = [0.234, 0.345].

Model 2, wave 2: Pooled R-Squared = 0.51, Pooled R-Squared C.I. = [0.468, 0.551].

Model 1, wave 3: Pooled R-Squared = 0.301, Pooled R-Squared C.I. = [0.249, 0.353].

Model 2, wave 3: Pooled R-Squared = 0.454, Pooled R-Squared C.I. = [0.405, 0.501].

Model 3, wave 3: Pooled R-Squared = 0.7, Pooled R-Squared C.I. = [0.648, 0.746].

Table 3 presents results of the multiple linear regression analysis in which each of the twelve disaster-related factors were analyzed for symptoms of distress at waves 2 and 3.

**Table 3:** **Associations between disaster-related factors and symptoms of distress at waves 2 and 3**^**a**^

	**Symptoms of distress at wave 2**						**Symptoms of distress at wave 3**								
	Model 1:			Model 2:			Model 1:			Model 2:			Model 3:		
	disaster-related factors			Disaster-related factors and symptoms distress and PTSD at wave 1			Disaster-related factors			Disaster-related factors and symptoms distress and PTSD at wave 1			Disaster-related factors and symptoms of distress and PTSD at waves 1 and 2		
	B	SE	P	B	SE	P	B	SE	P	B	SE	P	B	SE	P
Intercept	113.33	7.39	< .001	47.77	8.48	< .001	102.95	7.23	< .001	46.02	7.54	< .01	14.05	6.47	n.s.
House destroyed	12.62	4.24	< .05	2.63	3.73	n.s.	10.70	4.81	n.s.	2.04	4.45	n.s.	−0.22	2.97	n.s.
Injury self	17.16	7.37	n.s.	11.41	6.41	n.s.	16.39	6.44	< .05	11.37	5.82	n.s.	3.34	4.90	n.s.
Lost a loved one	10.15	7.42	n.s.	5.28	6.87	n.s.	10.61	6.93	n.s.	6.20	6.69	n.s.	2.50	4.63	n.s.
Witnessed injured victims	4.13	4.14	n.s.	3.25	3.65	n.s.	6.18	4.25	n.s.	5.46	4.01	n.s.	2.88	2.74	n.s.
Witnessed deceased victims	−2.96	5.15	n.s.	−7.13	4.72	n.s.	−11.08	5.98	n.s.	−14.93	5.62	< .05	−10.26	5.15	n.s.
Heard yelling individuals	3.72	4.01	n.s.	0.82	3.43	n.s.	5.61	3.95	n.s.	2.99	3.41	n.s.	2.4	3.00	n.s.
Felt heart palpitations	8.01	3.92	n.s.	−1.20	3.43	n.s.	6.08	4.08	n.s.	−2.16	3.59	n.s.	−1.51	2.82	n.s.
Felt intense anxiety	4.16	4.00	n.s.	1.04	3.52	n.s.	4.70	3.8	n.s.	1.77	3.46	n.s.	1.15	2.74	n.s.
Fled from explosions/ fire	−5.71	3.66	n.s.	−0.81	3.13	n.s.	−3.55	3.92	n.s.	0.72	3.60	n.s.	1.45	2.93	n.s.
Helped affected victims	2.49	3.4	n.s.	1.78	2.91	n.s.	4.60	3.43	n.s.	3.77	3.18	n.s.	2.41	2.50	n.s.
Felt feelings of guilt or shame	16.86	6.85	n.s.	−3.41	6.23	n.s.	22.40	6.56	< .05	4.76	6.00	n.s.	6.68	4.10	n.s.
Confusion	17.21	3.93	<.001	−5.15	3.88	n.s.	9.28	4.4	n.s.	−10.66	4.02	< .05	−6.90	3.65	n.s.
Symptoms of distress at wave 1				0.60	0.05	< .001				0.51	0.04	< .01	0.08	0.05	n.s.
Symptoms of PTSD at wave 1				−0.11	0.12	n.s.				0.004	0.12	n.s.	0.05	0.01	n.s.
Symptoms of distress at wave 2													0.70	0.05	< .01
Symptoms of PTSD at wave 2													0.12	0.13	n.s.

^a^ Multiple regression analysis, adjusted for sex, age, ethnicity, employment status, educational level, smoking/non-smoking and number of chronic diseases. N.s. = not significant.

Model 1, wave 2: Pooled R-Squared = 0.318, Pooled R-Squared C.I. = [0.266, 0.37].

Model 2, wave 2: Pooled R-Squared = 0.518, Pooled R-Squared C.I. = [0.477, 0.557].

Model 1, wave 3: Pooled R-Squared = 0.326, Pooled R-Squared C.I. = [0.278,0.374].

Model 2, wave 3: Pooled R-Squared = 0.47, Pooled R-Squared C.I. = [0.423, 0.515].

Model 3, wave 3: Pooled R-Squared = 0.708, Pooled R-Squared C.I. = [0.66, 0.754].

The disaster-related factors ‘house destroyed’ and ‘confusion’ were significant risk factors for a higher number of symptoms at wave 2 (model 1). After entering symptoms of distress and PTSD at wave 1 into the model these factors were no longer significantly associated with symptoms of distress at wave 2. The disaster-related factors ‘injury self’ and ‘feelings of guilt and shame’ were significant risk factors for a higher level of distress at wave 3 (model 1). After controlling for symptoms of distress and PTSD at wave 1 these factors were no longer significant predictors for symptoms of distress at wave 3. Interestingly, after controlling for symptoms of distress and PTSD at wave 1, ‘witnessed deceased victims’ (B = −14.93, SE = 5.62) and ‘confusion’ (B = −10.66, SE = 4.02) were associated with a lower level of symptoms of distress at wave 3. All associations were no longer significant when symptoms of distress and PTSD at wave 2 were entered into the model (model 3).

### Association between disaster exposure and symptoms of PTSD among survivors at waves 2 and 3

Table 4 presents the results of analyses with respect to the relation between the degree of disaster exposure (sum-score) and symptoms of PTSD. The degree of disaster exposure (sum-score) was related to a higher number of symptoms of PTSD at wave 2 (B = 2.23, SE = 0.19) and wave 3 (B = 2.19, SE = 0.20) (model 1). Although the association diminished after entering symptoms of distress and PTSD at wave 1 and 2 into the model, this association was still statistically significant (wave 2, model 2 and wave 3, model 3). Symptoms of PTSD and symptoms of distress were both significantly associated with a higher number of symptoms of PTSD at a later point in time.

**Table 4:** **Associations between disaster exposure (sum-score) and symptoms of ptsd at waves 2 and 3**^**a**^

	**Symptoms of PTSD at wave 2**						**Symptoms of PTSD at wave 3**								
	Model 1:			Model 2:			Model 1:			Model 2:			Model 3:		
	disaster-related factors			Disaster-related factors and symptoms distress and PTSD at wave 1			Disaster-related factors			Disaster-related factors and symptoms distress and PTSD at wave 1			Disaster-related factors and symptoms of distress and PTSD at waves 1 and 2		
	B	SE	P	B	SE	P	B	SE	P	B	SE	P	B	SE	P
Intercept	−23.56	4.06	< .01	−17.98	3.71	< .01	−32.63	4.13	< .05	−28.28	3.84	< .01	−20.59	3.67	< .05
Degree of disaster exposure	2.23	0.19	< .001	0.63	0.21	< .01	2.19	0.20	< .01	0.87	0.19	< .01	0.53	0.16	<.05
Symptoms of distress at wave 1				0.1	0.01	< .01				0.09	0.01	< .05	0.02	0.01	n.s.
Symptoms of PTSD at wave 1				0.24	0.04	< .01				0.19	0.05	< .01	0.07	0.04	n.s.
Symptoms of distress at wave 2													0.03	0.01	< .05
Symptoms of PTSD at wave 2													0.51	0.04	< .05

^a^Multiple regression analysis, adjusted for sex, age, ethnicity, employment status, educational level, smoking/non-smoking and number of chronic diseases. N.s. = not significant.

Model 1, wave 2: Pooled R-Squared = 0.245, Pooled R-Squared C.I. = [0.199, 0.292].

Model 2, wave 2: Pooled R-Squared = 0.403, Pooled R-Squared C.I. = [0.356, 0.449].

Model 1, wave 3: Pooled R-Squared = 0.307, Pooled R-Squared C.I. = [0.246, 0.368].

Model 2, wave 3: Pooled R-Squared = 0.413, Pooled R-Squared C.I. = [0.352, 0.473].

Model 3, wave 3: Pooled R-Squared = 0.594, Pooled R-Squared C.I. = [0.541, 0.643].

Table 5 shows the results of the multiple linear regression analysis in which each of the disaster-related factors were analyzed for symptoms of PTSD at waves 2 and 3. ‘House destroyed’, ‘felt heart palpitations’, ‘fled from explosions and/or fire’, ‘feelings of guilt and/or shame’, and ‘confusion’ were risk factors for symptoms of PTSD at wave 2, eighteen months post-disaster (model 1). After entering symptoms of distress and PTSD at wave 1 into the model, this effect diminished. In model 2, only the factor ‘house destroyed’ was independently associated with a higher level of symptoms of PTSD at wave 2 (B = 3.19, SE = 1.20).

**Table 5:** **Associations between disaster-related factors and symptoms of ptsd at waves 2 and 3**^**a**^

	**Symptoms of PTSD at wave 2**						**Symptoms of PTSD at wave 3**								
	Model 1:			Model 2:			Model 1:			Model 2:			Model 3:		
	disaster-related factors			Disaster-related factors and symptoms distress and PTSD at wave 1			Disaster-related factors			Disaster-related factors and symptoms distress and PTSD at wave 1			Disaster-related factors and symptoms of distress and PTSD at waves 1 and 2		
	B	SE	P	B	SE	P	B	SE	P	B	SE	P	B	SE	P
Intercept	4.94	2.31	n.s.	−9.71	2.56	<.01	−5.71	2.67	n.s.	−18.30	2.75	< .05	−14.80	2.73	< .05
House destroyed	5.44	1.25	< .01	3.19	1.20	<.05	5.66	1.27	< .05	3.73	1.25	< .05	2.03	1.05	n.s.
Injury self	2.27	2.11	n.s.	0.92	1.93	n.s.	2.86	2.05	n.s.	1.70	1.94	n.s.	0.90	2.00	n.s.
Lost a loved one	2.41	2.22	n.s.	0.82	2.04	n.s.	3.72	2.08	n.s.	2.39	1.92	n.s.	1.84	1.51	n.s.
Witnessed injured victims	2.54	1.30	n.s.	2.51	1.19	n.s.	2.98	1.29	n.s.	2.94	1.23	n.s.	1.58	1.02	n.s.
Witnessed deceased victims	3.56	1.74	n.s.	2.00	1.61	n.s.	−0.04	2.12	n.s.	−1.35	1.98	n.s.	−2.17	1.77	n.s.
Heard yelling individuals	1.17	1.39	n.s.	0.18	1.36	n.s.	1.76	1.28	n.s.	0.93	1.17	n.s.	0.81	1.03	n.s.
Felt heart palpitations	4.26	1.20	<.01	1.5	1.08	n.s.	2.14	1.10	n.s.	−0.19	1.05	n.s.	−0.92	0.90	n.s.
Felt intense anxiety	0.60	1.32	n.s.	−0.78	1.24	n.s.	1.26	1.25	n.s.	0.11	1.14	n.s.	0.48	0.96	n.s.
Fled from explosions and/or fire	−2.74	1.11	< .05	−1.6	1.01	n.s.	−1.95	1.18	n.s.	−0.97	1.08	n.s.	−0.13	0.92	n.s.
Helped affected victims	1.73	1.09	n.s.	0.98	0.99	n.s.	2.86	1.06	< .05	2.26	0.97	n.s.	1.71	0.81	n.s.
Felt feelings of guilt and/or shame	7.60	1.83	< .01	2.96	1.62	n.s.	6.87	2.49	< .05	2.89	2.42	n.s.	1.45	2.28	n.s.
Confusion	5.15	1.16	< .01	−1.40	1.24	n.s.	4.31	1.15	< .05	−1.23	1.26	n.s.	−0.35	1.25	n.s
Symptoms of distress at wave 1				0.1	0.01	< .01				0.09	0.01	< .05	0.02	0.02	n.s
Symptoms of PTSD at wave 1				0.26	0.04	< .01				0.21	0.05	< .05	0.08	0.04	n.s.
Symptoms of distress at wave 2													0.03	0.01	n.s.
Symptoms of PTSD at wave 2													0.51	0.04	< .05

^a^ Multiple regression analysis, adjusted for sex, age, ethnicity, employment status, educational level, smoking/non-smoking and number of chronic diseases. N.s. = not significant.

Model 1, wave 2: Pooled R-Squared = 0.282, Pooled R-Squared C.I. = [0.234,0.331].

Model 2, wave 2: Pooled R-Squared = 0.416, Pooled R-Squared C.I. = [0.37, 0.461].

Model 1, wave 3: Pooled R-Squared = 0.336, Pooled R-Squared C.I. = [0.274, 0.398].

Model 2, wave 3: Pooled R-Squared = 0.429, Pooled R-Squared C.I. = [0.365, 0.491].

Model 3, wave 3: Pooled R-Squared = 0.602, Pooled R-Squared C.I. = [0.549, 0.651].

The associations between the twelve disaster-related factors and symptoms of PTSD at wave 3 differed slightly from wave 2. ‘House destroyed’, ‘helped affected victims’, ‘feelings of guilt and/or shame’, and ‘confusion’ were positively associated with symptoms of PTSD at wave 3 (model 1). After entering symptoms of distress and PTSD at waves 1 and 2 into the model, none of the individual risk factors was independently related to symptoms of PTSD at wave 3 (model 3). Symptoms of PTSD and symptoms of distress at waves 1 and 2 were significantly associated with symptoms of PTSD at a later point in time.

### Association between disaster exposure and symptoms of PTSD at wave 4

Table 6 shows the association between the degree of disaster exposure and symptoms of PTSD among survivors at wave 4, ten years post-disaster. Since in wave 4 only native Dutch survivors participated, these models were run on this subgroup of the study population. At wave 4, ten years post-disaster, disaster exposure was no longer an independent risk factor for symptoms of PTSD after adjusting for symptoms at an earlier point in time (models 3 and 4). Symptoms of PTSD at eighteen months and four years post-disaster were significant independent predictors of symptoms of PTSD ten years post-disaster.

**Table 6:** **Associations between degree of disaster exposure (sum-score) and symptoms of ptsd at waves 2, 3 and wave 4 for native dutch survivors only**^**a**^

	**Symptoms of PTSD at wave 2**			**Symptoms of PTSD at wave 3**			**Symptoms of PTSD at wave 4**								
	Model 2:			Model 3:			Model 2:			Model 3:			Model 4:		
	Disaster-related factors and symptoms distress and PTSD at wave			Disaster-related factors and symptoms of distress and PTSD at wave 1 and 2			Disaster-related factors and symptoms distress and PTSD at wave 1			Disaster-related factors and symptoms of distress and PTSD at wave 1 and 2			Disaster-related factors and symptoms of distress and PTSD at wave 1, 2 and 3		
	B	SE	P	B	SE	P	B	SE	P	B	SE	P	B	SE	P
Intercept	−21.69	5.16	< .01	−21.93	4.33	< .01	−23.60	4.91	< .01	−15.85	5.0	< .05	−8.52	5.16	n.s.
Degree of disaster exposure	0.74	0.26	< .05	0.71	0.23	< .05	0.80	0.24	< .01	0.47	0.23	n.s.	0.19	0.23	n.s.
Symptoms of distress, wave 1	0.1	0.01	< .01	0.02	0.01	n.s.	0.07	0.02	< .01	0.02	0.02	n.s.	0.008	0.02	n.s.
Symptoms of PTSD, wave 1	0.30	0.04	< .01	0.09	0.04	n.s.	0.16	0.05	< .05	0.03	0.05	n.s.	−0.002	0.04	n.s.
Symptoms of distress, wave 2				0.005	0.01	n.s.				0.03	0.02	n.s.	0.02	0.02	n.s.
Symptoms of PTSD, wave 2				0.54	0.04	<.01				0.43	0.05	< .01	0.21	0.06	< .01
Symptoms of distress, wave 3													0.02	0.01	n.s.
Symptoms of PTSD, wave 3													0.39	0.07	< .01

^a^ Multiple regression analysis, adjusted for sex, age, ethnicity, employment status, educational level, smoking/non-smoking and number of chronic diseases. n.s. = not significant.

Model 2, wave 2: Pooled R-Squared = 0.365, Pooled R-Squared C.I. = [0.317, 0.412].

Model 3, wave 3: Pooled R-Squared = 0.544, Pooled R-Squared C.I. = [0.498, 0.587].

Model 2, wave 4: Pooled R-Squared = 0.21, Pooled R-Squared C.I. = [0.158, 0.266].

Model 3, wave 4: Pooled R-Squared = 0.378, Pooled R-Squared C.I. = [0.318, 0.437].

Model 4, wave 4: Pooled R-Squared = 0.464, Pooled R-Squared C.I. = [0.396, 0.53].

Because the individual disaster factors were not independently associated with symptoms of distress and PTSD at wave 3, we have chosen not to present the results for the relation between the individual disaster-related factors and symptoms of PTSD at wave 4.

## Discussion

This study shows a positive and independent relation between degree of disaster exposure and symptoms of PTSD until 4 years after the disaster. Ten years post-disaster, disaster exposure was no longer an independent risk factor for symptoms of PTSD. The variable ‘house destroyed’ was the only individual disaster-related factor that was independently related to symptoms to symptoms of PTSD until 18 months post-disaster. Disaster exposure (sum score or single experiences) was not an independent risk factor for symptoms of distress at any point in time. None of the individual disaster-related factors was independently related to symptoms of distress at waves 2 and 3. In contrast to our expectations, our findings, based on the pooled R squares, indicate that a sum score of disaster experiences did not improve the prediction of mental health problems compared to single disaster factors: they were almost equal.

In our study the degree of disaster exposure was an independent risk factor for symptoms of PTSD until four years post-disaster, but not for symptoms of distress. In an earlier study, Hobfoll et al. showed, in contrast to our results, that a sum-score of exposures to the WTC terrorist attacks was independently related to depression (as an indicator of distress), but not to symptoms of PTSD [29]. They did, however, not adjust for baseline symptoms. Since our study is the first that shows the independent relation between disaster exposure and symptoms of PTSD, while controlling for baseline symptoms, we can only speculate about the underlying mechanism. Since the SCL-90 asks for a broad range of (mental) health problems during the past week, the symptoms of distress reported by respondents in our study might be the result of life events, or daily stressors other than the disaster-related factors. Symptoms of PTSD (measured by the IES), on the other hand, measures symptoms of intrusion and avoidance, such as thoughts and dreams about the specific disaster and pictures of the disaster that comes into the respondents’ mind. This might explain why the degree of disaster exposure is independently related to symptoms of PTSD, but to symptoms of distress among survivors of the disaster. In addition, a somewhat similar pattern was found in a study among Iraq veterans: PTSD was related to war experiences, but general psychological distress was not related to these experiences [30].

After controlling for symptoms of distress and PTSD at earlier points in time, none of the individual disaster experiences were significantly related to symptoms of distress at waves 2 and 3. Remarkably, loss of a loved one was not significantly related to symptoms of distress and symptoms of PTSD either. While in other studies this factor increased the risk for these psychological problems significantly [5,31]. In our study, the variable ‘loss of a loved one’ included loss of family member, loss of a friend as well as loss of a colleague. Since only a small group of survivors (n = 19) lost a family member due to the disaster, we were not able to perform separate analyses on loss of family members. Possibly, loss of a family member might have been a significant, independent predictor in our study when it was analyzed as a separate factor.

After controlling for symptoms of distress and PTSD, ‘house destroyed’ was a independent risk factor for symptoms of PTSD at wave 2, 18 months after the disaster. The question remains why the variable “house destroyed” was the only specific disaster experience that was independently related to symptoms of PTSD 18 months post-disaster. This finding might be explained by the following. One major difference between the variable house destroyed and the other variables related to the disaster experiences is the permanent loss of (almost) all personal belongings. These personal belongings include for instance old photos, remembrances of (grand) mothers and fathers, and all other personal belongings that are/were significant to the survivors. In addition, being forced to relocate introduces new challenges to start a new life in a new neighborhood, introduces struggles with respect to financial compensations and related juridical problems. In contrast to the examined experiences during the disaster, these specific and objective circumstances may add additional stress over and above the traumatic stress related to the disaster experiences because these sources of stress continue to exist during the months and years after the event. In other words: the destruction on the house may cause more long lasting intense sources of stress while intense disaster experiences are relative acute and time limited. Survivors who’s house was not destructed did not have to deal with all these additional sources of stress.

Our findings clearly show that previous mental health problems were strong predictors of symptoms of PTSD and distress at subsequent waves, especially those during the wave before the current wave. The relation between disaster exposure and symptoms of distress and PTSD was mediated by symptoms of distress and PTSD at an earlier point in time.

Our results raises questions about how relations between some factors and mental health problems changed over time. For instance, Table 5 shows that the association between the factor “helped affected victims” and PTSD symptoms at wave 2 changed from not-significant (PTSD wave 2, model 1) to significant with respect to PTSD symptoms at wave 3 (PTSD wave 3, model 1). Since none of the factors were independently related to the outcomes, we refrain from speculating and have no clear answer to these questions.

This brings us to the issue whether or to what extent several disaster experiences in our study may be considered so-called “common factor-effect indicators” while other factors could perhaps be considered “composite causal indicators”, a topic that was explored and analyzed in the study of Layne and colleagues [32]. Although it would have been interesting to explore this in our study, assessing to what extent disasters experiences could be aggregated into dimensions according to their covariance structure (common factor indicators) while others according to their effects (composite causal indicators) was outside the aim of our study. In addition, assessing such type of indicators would require a large series of repeated analyses because it is it possible in principle that not all disaster experiences exclusively belong to one type of indicator, but that some belong to for instance the common factor type, while others may belong to the composite factor type (perhaps the number of required analyses may be limited by a theoretical framework enabling a pre-selection of relevant combinations). Further studies on this issue may help to improve or knowledge on risk factors for post-disasters mental health problems.

Another point that need to be mentioned is the use of Mental Health Services (MHS) utilization. Previous research has shown that part of the survivors of a disaster will seek of receiving treatment for their post-event mental health problems such as PTSD (symptomatology) that (hopefully) has a positive effect on their mental health status. The proportion of MHS users differs across disasters and time frame, depending on for instance the availability of MHS and needs of victims [33]. This indicates that, as in other disaster studies, that the associations between risk factors and post-event mental health problems may be affected by MHS use. Although we examined MHS use in our study sample [34-36], unfortunately our data does not enable further analyses on the effects of MHS use on the mental health of the respondents. Despite this, since our analyses - i.e. contrasting predictive values of separate items versus a sum score of items- are conducted within the same sample, comparison of outcomes are not inflated because of MHS use (i.e. is constant).

This study has some unique strengths. First, information about disaster exposure was collected three weeks post-disaster, which was a major advantage since delay in data collection may introduce recall bias and important data may be lost forever. Most studies on disasters only start to measure disaster-related factors several months to several years post-disaster [1].

Second, we examined symptoms of distress among survivors eighteen months and four years post-disaster, which gave us the opportunity to examine the predictive value of disaster-exposure for symptoms of distress in the medium term after a disaster. Besides this, we had the unique opportunity to collect data among a subgroup ten years after the disaster. Multiwave data of survivors of disasters, from 2–3 weeks up to four and ten years post-disaster is seldom available, so little is known about who is at risk of long-term psychological problems after disasters [1,8].

Besides these strengths, some potential limitations of this study should be considered. First, because only an estimated 30% of all affected residents participated in the first health survey, selective response and possible bias could be of concern in this study. The effect of selective participation at wave 1 on prevalence estimates (selection bias) was examined previously [13]. In this study the survey (questionnaire) data were combined with electronic medical records of residents’ general practitioners (GPs). Multiple imputations were used to examine the magnitude of selection bias on the prevalence estimates of self-reported health problems 3 weeks post-disasters. The results of this study showed that participants in the survey consulted their GPs for health problems in the year before and after the disaster more often than nonparticipants in at wave 1. Despite this selective participation, multiple imputations barely affected prevalence estimates of health problems in the survey 3 weeks post-disaster.

Also there was some selective response at the different waves of the longitudinal study. Those who completed the questionnaire at waves 1 to 3, were more likely to be female, middle-aged, highly educated, native Dutch and to have a paid job than those who did not complete all three waves. Analysis of Multiple Imputation (MI) showed that this selective response hardly affected the prevalence rates of health problems among survivors [20]. Despite this, we used MI in this paper to minimise any potential bias that may arise due to missingness [23,24].

Second, since we were primarily interested in the relation between disaster-related factors and symptoms of distress and PTSD, we did not examine psychological and social factors such as coping strategies, social support and the altered social context in our analysis. Although these factor might be confounders in the relation between exposure and health problems, it is not clear how these factors affect the relationship.

## Conclusion

Early identification of survivors at risk of psychological problems in the medium and longer term is important to target early intervention or treatment and to allocate the mental health resources after a disaster [1,3,4]. One way to early identify survivors that are at elevated risk of psychological problems is to distribute a concise questionnaire shortly after the disaster. The results of this study indicates that a concise questionnaire could provide valuable information about both disaster-related experiences and psychological problems of survivors, since disaster-related factors may increase the risk for psychological problems onset, which subsequently increase the risk for psychological problems on the medium and longer term. The results of this study also indicates that, besides the factor ‘house destroyed’, individual disaster-related factors are not independently related to symptoms of PTSD and distress on the longer term. For that reason, it seems to be useful to ask for a broad range of disaster-related experiences and use a sum-score when analyzing the questionnaire data.

Although the results need to be confirmed in studies that have a comparable design, the results of this study suggest that besides disaster exposure, psychological symptoms perpetuate the health problems of disaster survivors in the medium and long term. Health care workers should, therefore, aim their resources at those who still have psychological problems in the medium term after a disaster.

### Endnote

^a^based on our results for model variant (2c), we found it non-informative to present the results for model variant (2d).

### Ethical approval

The Medical Ethical Committee of the Netherlands Organisation for Applied Scientific Research (TNO, Zeist) approved the study protocols and all participants gave their written informed consent.

## Abbreviations

PTSD: Post-traumatic stress disorder; SCL-90-R: Symptom Checklist-90-Revised; IES: Impact of Event Scale; MICE: Multiple Imputations by Chained Equations; MI: Multiple Imputation.

## Competing interests

The authors declare that they have no competing interests.

## Authors’ contributions

BvdB was the lead author in writing the paper. AW analyzed the data for the present study and commented on the paper. PvdV, HB and LG commented on the analysis of the data and the writing of the paper. PvdV and LG designed the study and were the principal investigators of the first three waves. LG sought funding for the fourth wave, and planned and supervised the data collection of the fourth wave. All authors had full access to all data in the study and can take full responsibility for the integrity of the data and the accuracy of the data analysis. All authors read and approved the final manuscript.

## Pre-publication history

The pre-publication history for this paper can be accessed here:

http://www.biomedcentral.com/1471-244X/12/147/prepub
